# Enhanced Photon–Phonon Interaction in WSe_2_ Acoustic Nanocavities

**DOI:** 10.1021/acsphotonics.3c01601

**Published:** 2024-03-07

**Authors:** Alex D. Carr, Claudia Ruppert, Anton K. Samusev, Giulia Magnabosco, Nicolas Vogel, Tetiana L. Linnik, Andrew W. Rushforth, Manfred Bayer, Alexey V. Scherbakov, Andrey V. Akimov

**Affiliations:** † School of Physics and Astronomy, University of Nottingham, Nottingham NG7 2RD, United Kingdom; ‡ Experimentelle Physik 2, Technische Universität Dortmund, Otto-Hahn-Str. 4a, 44227 Dortmund, Germany; § Institute of Particle Technology, Friedrich-Alexander-Universität Erlangen-Nürnberg, Cauerstr. 4, 91058 Erlangen, Germany; ∥ Department of Theoretical Physics, V.E. Lashkaryov Institute of Semiconductor Physics, 03028 Kyiv, Ukraine

**Keywords:** coherent phonons, exciton−phonon interaction, van der Waals nanolayers, transition metal dichalcogenides

## Abstract

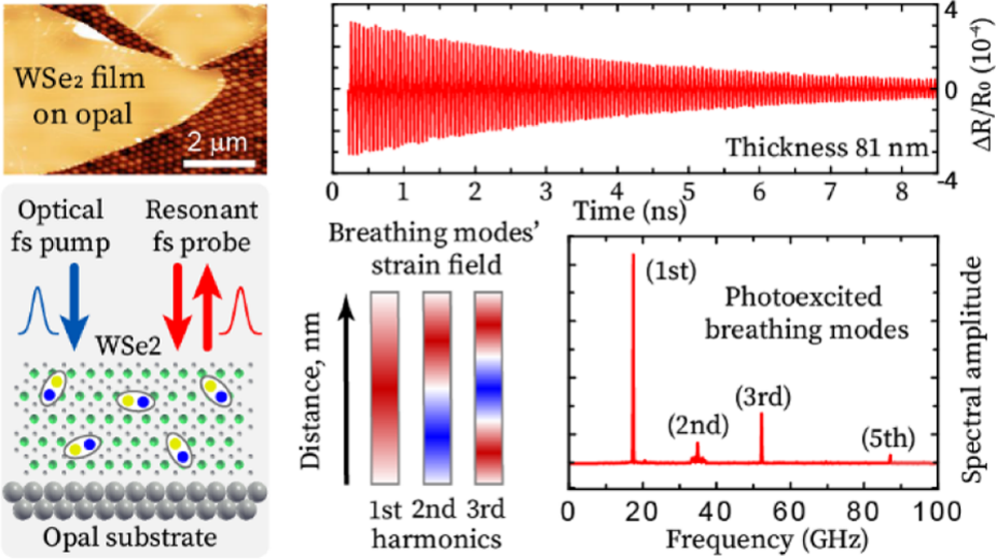

Acoustic nanocavities (ANCs) with resonance frequencies much above 1 GHz are prospective to be exploited in sensors and quantum operating devices. Nowadays, acoustic nanocavities fabricated from van der Waals (vdW) nanolayers allow them to exhibit resonance frequencies of the breathing acoustic mode up to *f* ∼ 1 THz and quality factors up to *Q* ∼ 10^3^. For such high acoustic frequencies, electrical methods fail, and optical techniques are used for the generation and detection of coherent phonons. Here, we study experimentally acoustic nanocavities fabricated from WSe_2_ layers with thicknesses from 8 up to 130 nm deposited onto silica colloidal crystals. The substrate provides a strong mechanical support for the layers while keeping their acoustic properties the same as in membranes. We concentrate on experimental and theoretical studies of the amplitude of the optically measured acoustic signal from the breathing mode, which is the most important characteristic for acousto-optical devices. We probe the acoustic signal optically with a single wavelength in the vicinity of the exciton resonance and measure the relative changes in the reflectivity induced by coherent phonons up to 3 × 10^–4^ for *f* ∼ 100 GHz. We reveal the enhancement of photon–phonon interaction for a wide range of acoustic frequencies and show high sensitivity of the signal amplitude to the photoelastic constants governed by the deformation potential and dielectric function for photon energies near the exciton resonance. We also reveal a resonance in the photoelastic response (we call it photoelastic resonance) in the nanolayers with thickness close to the Bragg condition. The estimates show the capability of acoustic nanocavities with an exciton resonance for operations with high-frequency single phonons at an elevated temperature.

## Introduction

Nanodevices with acoustic resonances (i.e., nanoacoustic devices) in the frequency range *f* = 1–10 gigahertz (GHz) have shown their capability to perform quantum operations^1−3^ and optical cooling^4^ and serve as filters,^5^ modulators,^6^ and sensors.^7^ The challenging task for exploiting nanoacoustic devices in quantum technologies and communications is to extend the frequency range of acoustic resonances above 10 GHz up to the terahertz (THz) range. This will increase the speed and sensitivity of nanoacoustic devices and enable performing quantum operations without the need for ultralow temperatures. Acoustic nanocavities (ANCs), which localize longitudinal acoustic (LA) phonons, are one of the devices, which could perform these functions. Examples of high-frequency ANCs include superlattices, which localize phonons with frequencies lying in the acoustic stop-bands,^8,9^ and membranes fabricated from various semiconductors (e.g., Si,^10,11^ GaAs,^12^ GaN^13^). Unique sub-THz and THz acoustic properties have been revealed in nanolayer-related heterostructures fabricated from van der Waals (vdW) materials like graphene,^14^ transition metal dichalcogenides (TMDs),^15−21^ and others.^22,23^ VdW nanolayers, which are easily fabricated by exfoliation techniques, enable ANCs to reach *f* ∼ 1 THz and quality factor *Q* > 10^3^ so that *f* × *Q* reaches the record value of 10^14^ Hz^17,18^ (for lower frequency nanomechanical properties, see review^24^). Such high finesse at extremely high acoustic frequencies leads to a new paradigm in the engineering of high-frequency communication and quantum devices.

In the previous studies^14−23^ of vdW-based ANCs, the main task was to increase the frequency and decay time of the generated coherent LA acoustic phonons while the efficiency of their generation and detection did not get much attention. Nevertheless, the amplitude of the signal induced by coherent phonon oscillations generated and detected in ANCs is the most important parameter for exploiting vdW ANCs in practical devices. The specific feature of all high-frequency ANCs is the inevitability of using optical techniques for the generation and detection of localized LA acoustic phonons because electrical methods which use piezoelectric transducers nowadays fail at *f* > 20 GHz.^25^ A great challenge is to achieve generation and detection efficiencies high enough for unambiguous recognition of a single phonon quantum, which requires high sensitivity of the optical response to the generated acoustic field. Then, the optimization of the vdW ANCs requires an understanding of the photon–phonon interaction in the vdW nanolayers.

In the present paper, we study ANCs fabricated from WSe_2_ nanolayers. WSe_2_ is a vdW TMD material with well-known optical and elastic properties. Using optical generation and detection of coherent LA phonons with frequencies *f* of the breathing mode from 10 up to ∼200 GHz, we concentrate our studies on the amplitude of the detected signal as a function of the nanolayer thickness and, respectively, the fundamental resonance frequency and its higher harmonics. Experimentally, we observe relative changes in the reflectivity of more than 10^–4^, which is an order of magnitude higher than reported in vdW ANCs studied previously.^17,18^ The enhancement of the coherent signal amplitude measured in our experiments is explained by the essential role of the exciton resonance, which boosts photon–phonon interaction in the WSe_2_ layers. The effects of the strong exciton–phonon interaction for coherent phonons have been observed in traditional semiconductor nanostructures (for reference, see review^26^) and studied intensively in vdW nanolayers for noncoherent phonons in Raman^27−30^ and photoluminescence^31,32^ experiments. In the analysis of our experimental results, our theoretical model reveals a strong dependence of the coherent signal amplitude on the complex photoelastic constant when probing with the wavelength in the vicinity of the exciton resonance in WSe_2_. When the thickness of the layer is close to the optical Bragg condition, we experimentally observe a resonance in the photoelastic response (i.e., a photoelastic resonance).

## Results and Discussion

**Figure 1:**
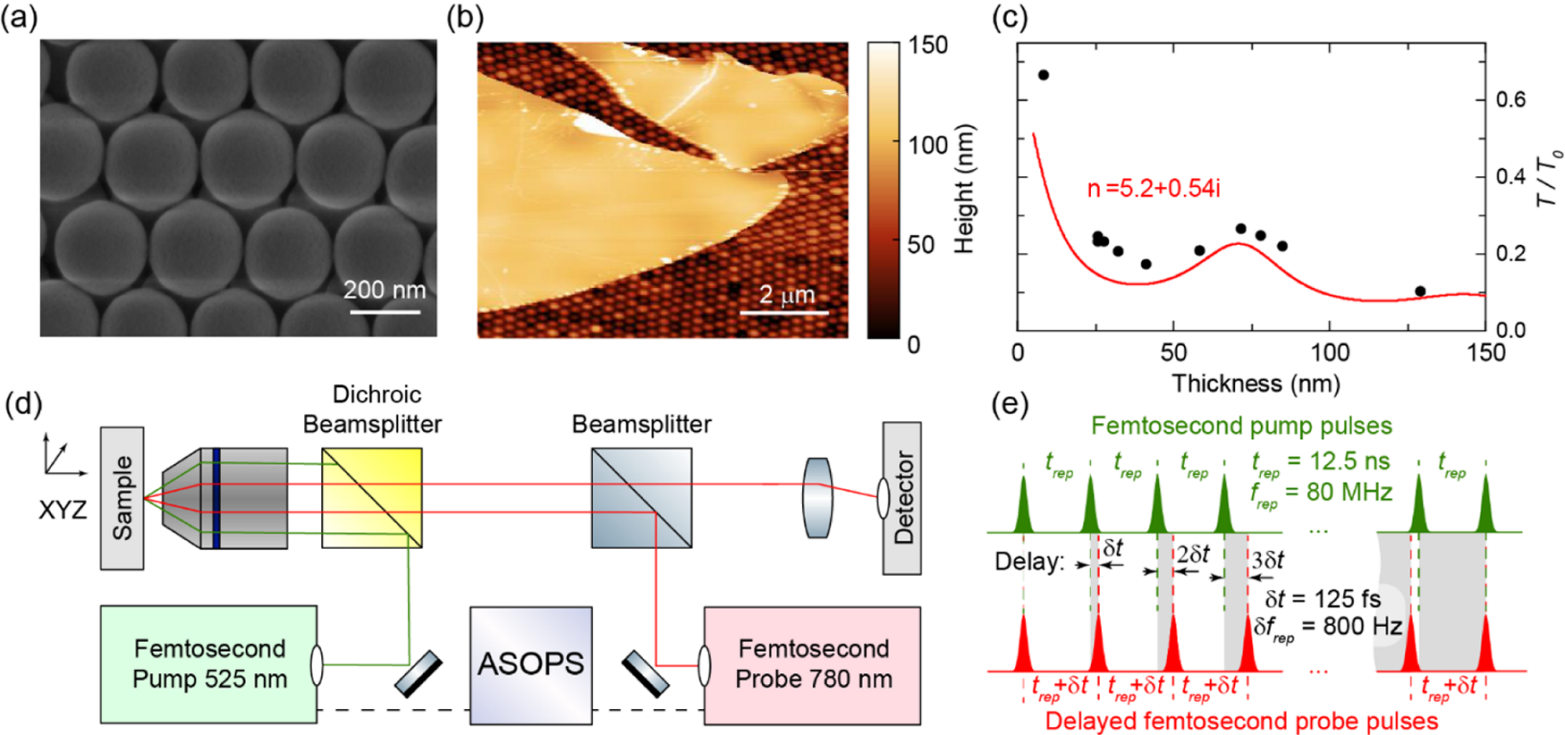
(a) Scanning electron microscopy (SEM) image of the bare opal film. (b) Atomic force microscopy (AFM) image of the WSe_2_ layer with the thickness *d* = 60 nm transferred on the opal film. (c) The measured (squares) and calculated (solid line) normalized transmission of the probe light (λ = 780 nm) through the WSe_2_ nanolayers on opals as a function of layer thickness. *T*_0_ corresponds to the transmission through the bare opal film. (d) Experimental setup based on an asynchronous optical sampling (ASOPS). (e) The repetition rates of both pump and probe lasers are *f*_*rep*_ ≈80 MHz. The ASOPS module allows for precise stabilization of the difference in the repetition rates at a value of δ*f*_rep_ = 800 Hz. This enables scanning of the delay between pump and probe pulses with an incremental step of δ*t* = 125 fs.

We study WSe_2_ nanosheets exfoliated from bulk WSe_2_ provided by HQ Graphene and transferred onto a layer of self-assembled silica colloidal particles with a diameter of 265 nm, which form a close-packed colloidal crystal, known as opal structure^33^ (for details, see the Methods section). The scanning electron microscopy (SEM) and atomic force microscopy (AFM) images of the opal layer without and with the transferred WSe_2_ nanolayer are shown in Figure 1a,b, respectively. The opal layer plays the role of a firm mechanical and thermal support for the nanolayers. In contrast to the vdW layers on plane substrates, WSe_2_ nanosheets on opals possess LA phonon properties similar to suspended layers, which prevents the escape of phonons into the substrate. As will be shown below, the ANCs on the silica opal films have values of the lifetime similar to those of the suspended layers.

The symbols in Figure 1c show the measured optical transmission *T*/*T*_0_ of light with λ = 780 nm through the nanolayer+opal as a function of layer thickness *d* (*T*_0_ is the transmission through the bare opal film on the SiO_2_ substrate). The solid curve in Figure 1c is calculated using the equation for light transmission through a film on a substrate^34^ using the complex refractive index for multilayer WSe_2_*n* = 5.26 + 0.54*i*.^35,36^ The similar shapes and close values for *T*/*T*_0_ in the experimental and calculated curves allow us to use the same value of *n* as measured earlier for the analysis.^35,36^

The experimental scheme for the generation and detection of coherent phonons in ANCs is shown in Figure 1d. Coherent LA phonons in WSe_2_ ANCs are generated and detected optically using an 80 MHz ASOPS^37^ pump–probe technique with the offset 800 Hz and fixed central wavelength (see the scheme in Figure 1e). The pump (wavelength Λ = 525 nm) and probe (wavelength λ = 780 nm) laser pulses (both 150 fs duration and average power ∼0.3 mW) are focused on a spot on the nanolayer with a diameter of 1.5 μm.

**Figure 2:**
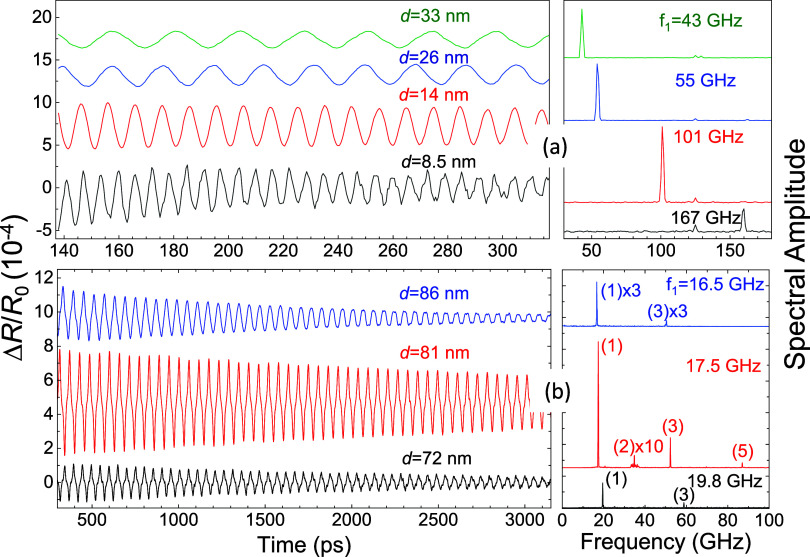
Fragments of the measured temporal signals (left panels) and their fast Fourier transforms (FFTs, right panels) for thin (a) and thick (b) WSe_2_ layers. The FFTs are obtained for the full time interval. In (a), the signals include only the fundamental harmonic; in (b), several acoustic harmonics are detected; the harmonic number, *s*, is shown in parentheses in the right panel of (b). The values of the fundamental frequency are shown for each signal in the right panels. The spectral line at *f* = 125 GHz in the right panel (a) corresponds to the parasite ASOPS interference (see the Methods section).

In the experiments, we measure 42 WSe_2_ ANCs with thicknesses *d* between 8 and 130 nm (for details, see the Methods section). Typical examples of the measured pump–probe reflectivity temporal signals Δ*R*(*t*)/*R*_0_ (*R*_0_ is the reflectivity of the probe beam without pump excitation) for 7 ANCs of various thicknesses are shown in the left panels of Figure 2a,b after the subtraction of the slowly decaying “electronic” background. It is seen that the signals show oscillations with the amplitude Δ*R*/*R*_0_ ∼ 1 × 10^–4^. The right panels of Figure 2a,b are fast Fourier transforms (FFTs) of Δ*R*(*t*)/*R*_0_ performed in the entire available temporal window of 12.5 ns. The FFTs consist of narrow spectral lines, which correspond to the acoustic frequency *f*_1_ of the fundamental breathing mode in ANC and higher *s*-harmonics. For the ANCs with *f*_1_ > 25 GHz, the fundamental (*s* = 1) harmonic dominates in the spectra, as demonstrated in Figure 2a. In the signals measured in the ANCs with a lower fundamental frequency and shown in Figure 2b, higher harmonics with frequencies *f_s_* can also be seen.

**Figure 3:**
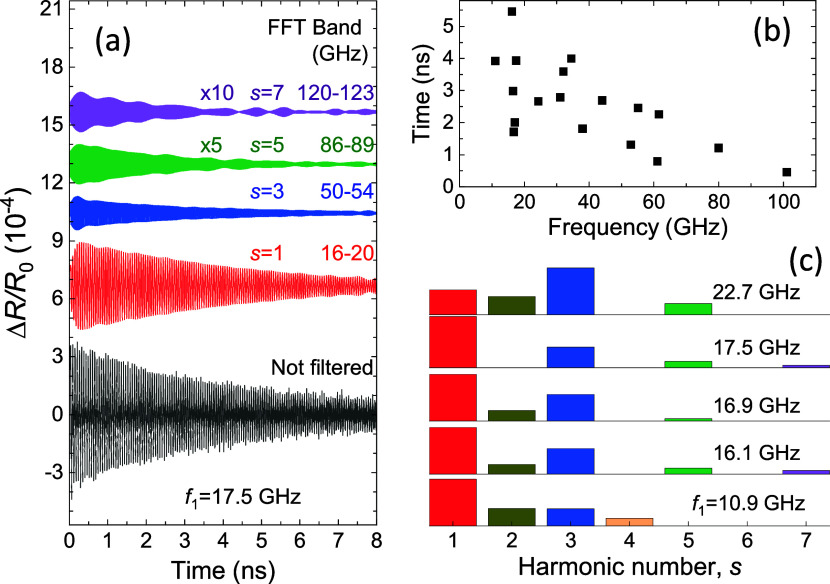
(a) Examples of filtered signals obtained by FFT filtering of the measured signal (lower curve) in the frequency bands around the central frequency of the corresponding harmonic. Filtered signals are used for fitting with a decaying harmonic function in order to obtain the decay time and amplitude of each harmonic. (b) Dependence of the decay time of the fundamental harmonic as a function of its frequency. (c) Normalized harmonic amplitude as a function of harmonic number for several fundamental frequencies.

The amplitudes *A*_*s*_ and decay times τ_*s*_ for the *s*-th harmonics are obtained by filtering the experimental temporal signals with bandpass filters around *f*_*s*_ and fitting the filtered signals using the equation: Δ*R*(*t*)/*R*_0_ = *A*_*s*_*e*^–*t*/τ^_^s^_ sin ω_*s*_*t*, where ω*_s_* = 2π*f*_*s*_. As an example, the filtered signals for the ANC with *f*_1_ = 17.5 GHz are shown in Figure 3a. Figure 3b shows the measured dependence of the decay time τ_1_ on the fundamental phonon mode with the frequency *f*_1_. In agreement with earlier experimental studies^17,18^ performed on suspended vdW ANCs, τ_1_ gradually decreases from values of several nanoseconds at *f*_1_ ∼ 10 GHz to τ_1_ < 1 ns at *f*_1_ ∼ 100 GHz. At this frequency interval, the decay is governed by surface roughness and in-plane escape of the acoustic waves from the optically excited area.^17,18^ The exfoliation technique does not control the roughness, which varies for different nanolayers, resulting in a large scatter of τ_1_. The agreement of the measured τ_1_ with the values measured in the previous experiments performed on suspended ACNs^17,18^ fabricated from TMDs allows us to consider the elastic properties of the ANCs on opals to be similar to suspended layers and use the well-known relation for the resonant frequency *f*_*s*_ with out-of-plane LA sound velocity *v* and layer thickness *d*
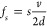
1

For obtaining *v*, we measured the thickness of several nanolayers by AFM before the transfer onto the opal layer, and using eq 1, we get *v* = 2840 ± 20 ms^–1^, which is 12% higher than measured in earlier experiments.^38^ Likely, this difference is due to the different bulk WSe_2_ materials used in our experiments.

Figure 3c shows the dependence of the normalized amplitudes *A*_*s*_ of higher harmonics on the fundamental frequency *f*_1_ of the breathing mode. It is seen that the higher harmonics contribute to the measured temporal signals on par with the fundamental harmonic and, in some cases, even have a larger amplitude (for example, the third harmonic at *f*_1_ = 22.7 GHz).

**Figure 4:**
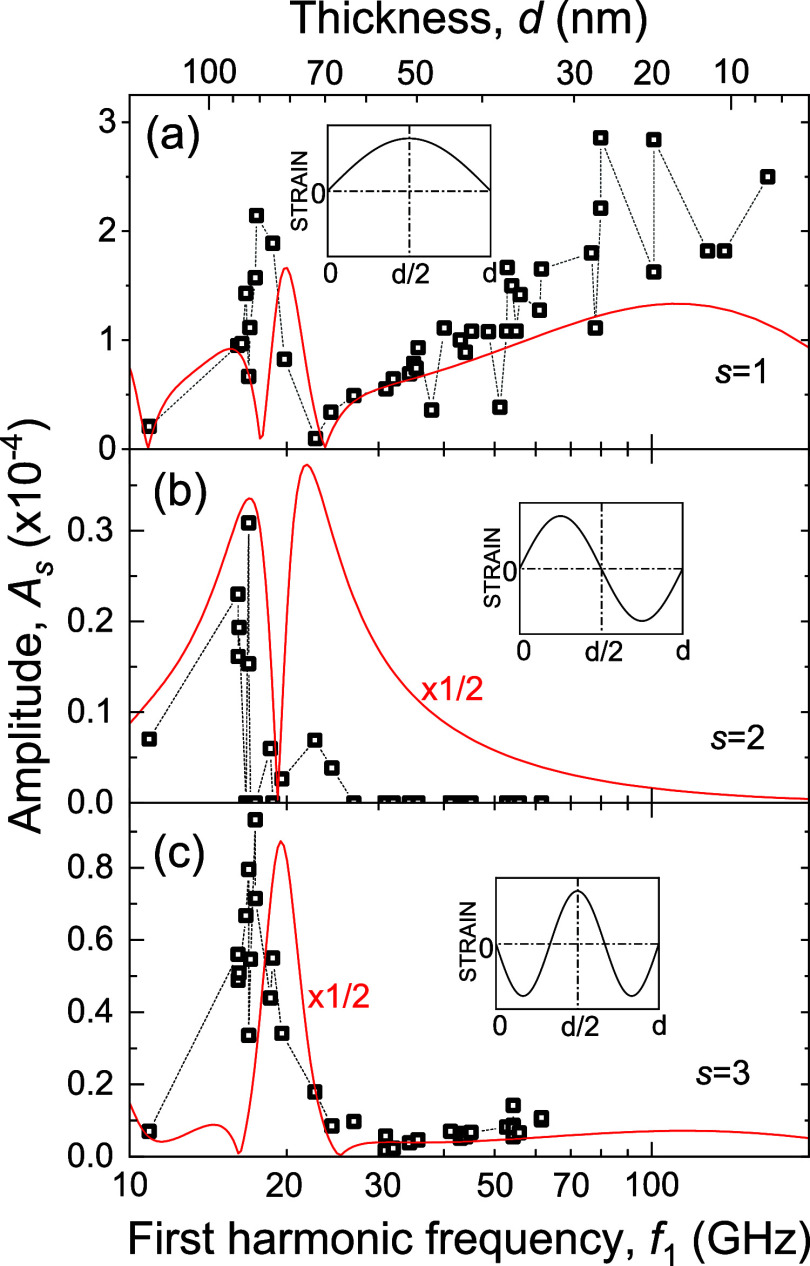
Measured (symbols) and calculated (solid line) dependences of the amplitude of the 1st (a), 2nd (b), and 3rd (c) harmonics of the measured signal as a function of the fundamental frequency *f*_1_ (lower scale) and layer thickness (upper scale). The value of deformation potential Ξ = −1 eV and fitting parameter *D* = 0.5 eV in the simulations are chosen to get the best quantitative agreement between the theory and experiment for the 1st harmonic. The insets show the spatial distribution of strain for the corresponding acoustic harmonics.

The symbols in Figure 4 show the dependence of the amplitude values *A*_*s*_ on *f*_1_ (lower scale) and on *d* (upper scale) for Δ*R*(*t*)/*R*_0_ obtained in all measured ANCs. It is seen that at *f*_1_ ≈ 18 GHz, there is a local maximum in the measured dependences *A*_1_ (*f*_1_) [panel (a)] and *A*_3_(*f*_1_) [panel (c)]. The ANC with *f*_1_ = 18 GHz has thickness *d* = 79 nm, which is very close to the thickness corresponding to the Bragg condition for normal incidence of the probe light, *d*_B_ = λ/(2*Re*{*n*}) = 74 nm. The correlation of the maximum position in Figure 4 and the Bragg law points to a photoelastic resonance in the studied ANCs. For *f*_1_ > 22 GHz, the measured dependence *A*_1_ (*f*_1_) shows (see Figure 4a) a gradual increase of *A*_1_ with the increase of *f*_1_ and a corresponding decrease of *d*. The higher harmonics (see Figure 4b,c) do not show such an increase, and their amplitude gradually decreases with the increase of *f*_1_. We observe quite a high scatter (it may reach 100%) of the measured points, which will be explained below by the strong sensitivity of *A*_*s*_ to the photoelastic parameters in WSe_2_ in the vicinity of the exciton resonance.

To analyze the amplitude of the measured acoustic signal in the studied ANCs, we consider two theoretical tasks. The first is the calculation of the dynamical strain at the resonance frequency given by eq 1 and generated by optical pump pulses. The second task is the simulation of the reflected probe optical pulse and obtaining the amplitude *A*_*s*_ of the detected signal at resonance frequency *f*_*s*_. The theoretical background for both tasks was developed in earlier works,^39−41^ and here, we calculate the values for *A*_*s*_ and their dependencies on *f*_*s*_ in order to compare them with the experimentally measured dependences on layer thickness and validate the given approach for designing efficient ANCs.

The calculation of strain η_*zz*_(*t*, *z*) = ∂*u*/∂*z* starts with solving the one-dimensional elastic equation for the displacement *u*(*t*, *z*) of atoms in the ANC, where *t* is the time and *z* is the coordinate normal to the plane of the layer
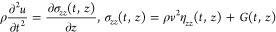
2with boundary conditions of zero stress at the free surfaces: σ_*zz*_(*t*, *z*) = 0 at *z* = 0 and *z* = *d*, ρ = 9.32 g/cm^3^ is the mass density of WSe_2_, and *G*(*t*, *z*) is the stress generated by an ultrashort optical pump pulse applied at *t* = 0^39^
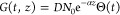
3where *N*_0_ is the density of excited electron–hole pairs at the surface and Θ(*t*) is the unity step function. Using the refractive index 4.6 + 1.4*i*^35,36^ for the pump wavelength Λ = 525 nm and assuming the absence of ultrafast carrier diffusion and recombination, we get α = 3.3 × 10^7^ m^–1^ and correspondingly the density of electron–hole pairs at the surface *N*_0_ = 7.3 × 10^25^ m^–3^ for the used fluence 0.15 mJ/cm^2^. The coefficient *D* has dimensions of energy and depends on the stress generation mechanism. The main mechanisms are the deformation potential and thermoelastic effects.^39,41^ It is difficult to estimate the value *D* because the parameters (deformation potential and linear expansion coefficient) are not known for WSe_2_. In the present analysis, we leave *D* as a fitting parameter, which allows us to describe well the absolute amplitude *A*_*s*_ at the final stage of the simulations.

The analytical solution of eq 2 for the strain components of the *s*-th harmonic may be written as
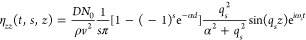
4where *q*_*s*_ = ω*_s_*/*v* is the wave vector of the *s*-th resonance mode.

**Figure 5:**
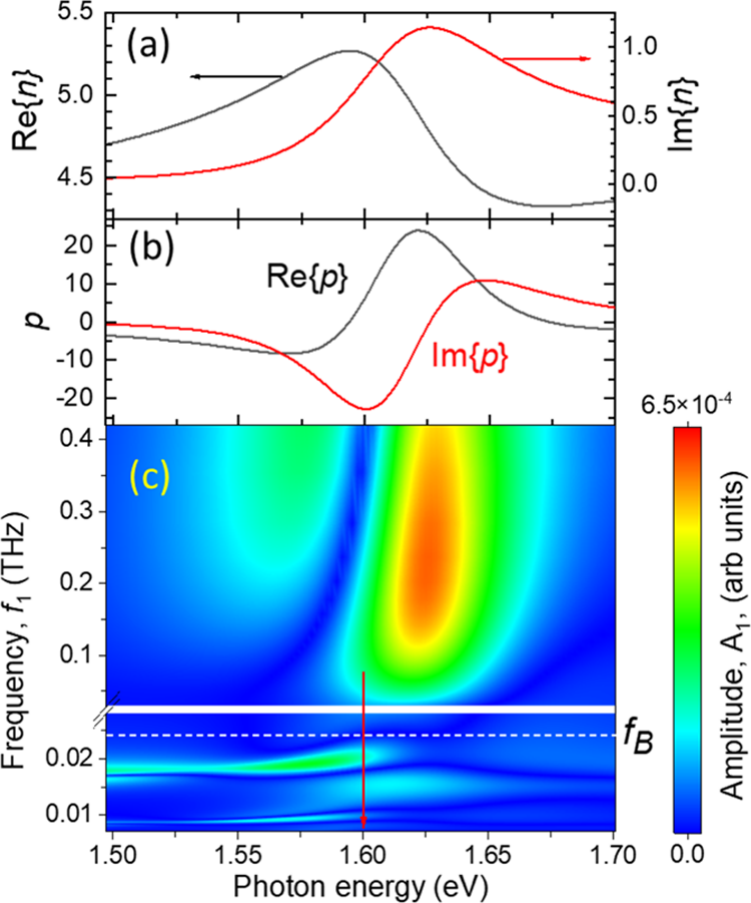
Dependences of the complex refractive index, *n ,* (a) and reduced photoelastic constant, *p*, (b) on the photon energy in the vicinity of the direct exciton resonance in multilayer WSe_2_. The data for plotting (a) is taken from ref (36) (c). Contour plot of the amplitude *A*_1_ of the first harmonic on the probe photon energy calculated for *D* = 1 eV (with different vertical scales in the lower and upper parts); vertical arrow indicates the photon energy of the probe light used in the experiments.

To calculate the reflectivity changes Δ*R*(*t*)/*R*_0_, we use the theoretical approach developed earlier,^40^ where it is shown that Δ*R*(*t*)/*R*_0_ may be written as
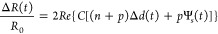
5where *p* = d*n*/dη_*zz*_ is the reduced photoelastic constant, Δ*d*(*t*) = *u*(*d*) – *u*(0) is the change of the layer thickness due to generated stress, *C* is a complex number, which depends on the optical parameters of the ANC, and Ψ_*s*_(*t*) is a function, which depends on the spatial distribution of the generated strain given by eq 4 and the optical parameters of the ANC. The equations for *C* and Ψ_*s*_(*t*) are given in the Methods section. Equations 4 and 5 are used to calculate the amplitude *A*_*s*_ of the corresponding *s*-th harmonic. There are two contributions to the signal Δ*R*(*t*)/*R*_0_: the first term in eq 5 includes both *n* and *p* and is governed by the modulation of the layer thickness Δ*d*(*t*) and the second term in eq 5 is governed only by the photoelastic effect in the bulk of the layer. The probe wavelength lies in the vicinity of the direct exciton resonance of multilayer WSe_2_, where strong dispersion takes place.^35^ Then, the value of *p* may be written as
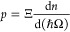
6where Ξ is the out-of-plane deformation potential (Ξ = d*E*_exc_/dη_*zz*_, *E*_exc_ is the direct exciton energy) and ℏΩ is the energy of the optical probe quantum. For multilayer WSe_2_, we estimate the value of the out-of-plane deformation potential from the experimentally measured dependence of direct exciton energy on hydrostatic pressure^42^ (for details, see the Methods section) and d*n*/d(ℏΩ) may be calculated from the dispersion of multilayer WSe_2_.^35,36^Figure 5a,b shows the dependences of the real and imaginary parts of *n* and *p* on ℏΩ in the vicinity of the direct exciton resonance for Ξ = −1 eV. It is seen that *n* and *p* depend strongly on ℏΩ, and for a certain ℏΩ, the values of *p* may essentially exceed *n*, resulting in a leading role of the photoelastic effect when probing in the vicinity of the exciton resonance. The value of *p* in the vicinity of the exciton resonance is much higher than that for nonresonant ℏΩ and this fact is the main reason for the enhancement of photon–phonon interaction in the whole phonon frequency range.

Figure 5c is a contour plot of the calculated *A*_1_(*f*_1_) for various ℏΩ. It is seen that the dependence of *A*_1_(*f*_1_) is very sensitive to ℏΩ. In the dependence *A*_1_(*f*_1_), we may distinguish two frequency ranges separated in Figure 5c by the dashed line at *f_B_* ∼ 25 GHz (mind also different vertical scales in lower and upper parts of Figure 5c): (1) *f*_1_ < *f*_*B*_ and (2) *f*_1_ > *f_B_*. The physical nature of *f_B_* is associated with the Bragg condition for normal incidence when the layer thickness *d*_B_ = λ*m*/2*Re*{*n*}, where *m* = 1, 2,··· (for *m* = 1 *d*_B_ = 74 nm). The corresponding first harmonic acoustic resonance frequency is equal to *f*_1_ = 19.2 GHz. We choose the value of *f_B_* = 25 GHz, which is beyond the wings related to the resonances *A*_*s*_(*f*_1_) according to the Bragg condition. It is seen that for *f*_1_ < *f_B_*, the interference of the probe light on the ANC’s surfaces results in photoelastic resonances in the calculated *A*_1_(*f*_1_), the positions of which depend on *n* and *p* and correspondingly on ℏΩ. In the frequency range *f*_1_ > *f*_*B*_, which is well above the Bragg conditions, *A*_1_(*f*_1_) does not show narrow peaks and, depending on ℏΩ, possesses a gradual increase or decrease with the increase of *f*_1_. Finally, *A*_1_ decreases for nanolayers with a thickness of several monolayers and correspondingly *f*_1_ ∼ 1 THz (not shown in Figure 5c).

To compare the simulations with the experiment, we present the calculated curves *A*_*s*_(*f*_1_) for ℏΩ = 1.6 eV together with the experimental points in Figure 4 for *s* = 1 [panel (a)], *s* = 2 [panel (b)], and *s* = 3 [panel (c)]. Both experimental and calculated dependences show peaks and nodes for *f*_1_ < *f*_*B*_. *A*_1_ gradually increases with the increase of *f*_1_ for *f*_1_ > *f*_*B*_, while the amplitudes *A*_*s*_ of higher harmonics show the decrease. To obtain a quantitative agreement in the absolute values for the amplitudes *A*_1_, we use Ξ = −1 eV (see the Methods section) and *D* = 0.5 eV in the calculations, which are reasonable values for WSe_2_.^42^ We explain the strong scatter (up to 100%) of the experimental points by the strain that appears due to contact of the ANCs with the opal surface. For instance, the strain ∼1% shifts the exciton resonance on the order of 30 meV, changing *n* and *p* and correspondingly *A*_*s*_.

Comparing the measured and calculated amplitudes of higher harmonics (*s* = 2 and *s* = 3, see Figure 4b,c, respectively), we see that experimentally measured values of *A*_*s*_ are a factor of 2 lower than predicted by theory. We think that the reason for this is that a spatial profile of optically generated stress differs from the exponentially decaying profile used in eq 3. Indeed, the amplitudes of higher harmonics are very sensitive to the spatial overlap of the acoustic mode profile (see insets in Figure 4) with the profiles of the generated stress (see eq 3) and also the optical field (see eq 5). For instance, symmetric acoustic modes (*s* = 2, 4, 6, etc.) are not excited for uniform excitation. The qualitative modeling of the spatial profile effects is given in Supporting Information 1. For the detailed discussion on the higher harmonic generation, we refer to the studies of coherent phonons in semiconductor membranes.^43^

Summarizing the comparison of the experimental data and theoretical calculations, we point at the following qualitative agreement: (i) the dependences of the measured amplitude on the fundamental frequency show peaks and dips when the layer thickness is close to the Bragg condition for the probe light; (ii) the measured amplitude of the fundamental acoustic harmonic gradually increases with the increase of breathing mode frequency up to *f*_1_ ∼ 100 GHz (which corresponds to the decrease of the layer thickness down to *d* ∼ 10 nm); and (iii) the amplitudes *A*_*s*_ of the higher acoustic harmonics for the thicknesses *d* ∼ 100 nm are comparable with the amplitude *A*_1_ of the fundamental harmonic. Among few quantitative disagreements between the experiment and theory, we point at a slight shift (∼10%) toward the low frequency of the experimental photoelastic peaks presented in Figure 4. The most likely reason for this is the variation of the photoelastic constant due to the built-in strain induced by the contact of the layer with the opal substrate and possible heating from the optical excitation. Some deviations may also come from not including the finite body angle of the focused probe beam in the simulations.

Finally, we discuss the prospect of using vdW ANCs as the elements of quantum devices operating with single phonons. In the experiments, the amplitudes of the detected *s* = 1 resonance signals *A*_1_ ∼ *S*η_*zz*_, where *S* is the sensitivity of the detection and η_*zz*_ is the amplitude of strain in the middle of the layer. For the probe beam used in the described experiments, *S* ∼ 10. The estimates show that for the used pump fluence, the number of generated phonons at the first resonance with *f*_1_ = 100 GHz is *N*_*p*_ ∼ 10^4^ phonons/μm^2^ and the measured amplitude is *A*_1_ ∼ 10^–4^. The amplitude *A*_1_ is proportional to the strain amplitude η_*zz*_ and thus proportional to . Then, decreasing the pump fluence by 2 orders of magnitude, we would get *A*_1_ ∼ 10^–6^ and *N*_p_ ∼ 1 phonon/μm^2^. The value *A*_1_ ∼ 10^–6^ can be technically measured nowadays in pump–probe experiments, and correspondingly, it is possible to measure single phonons if detected in the vicinity of the exciton resonance.

We have studied coherent vibrations of high-frequency acoustic nanocavities fabricated from van der Waals layers of WSe_2_ with thicknesses from 8 up to 139 nm. The layers are stamped on opal films formed by the self-assembly of silica colloidal particles, which play the role of acoustic isolators for high-frequency phonons. In contrast to the substrates with microholes fabricated using electron beam lithography, the substrates with colloidal particles cover a large area (up to several cm^2^) and make integration into devices (e.g., optical fibers and integration circles) much easier.^44^ Using an ultrafast optical pump–probe technique with a probe wavelength in the vicinity of the direct exciton resonance in WSe_2_, we get an amplitude of the coherent optical reflectivity modulation as high as 3 × 10^–4^ for frequency ∼100 GHz, which is an order of magnitude higher than detected in previous experiments with van der Waals ANCs.^17,18^ Theoretical analysis of the optical response to coherent acoustic excitation shows that the efficiency of the photoelastic effect for probe photon energy close to the exciton resonance increases enormously, leading to the enhanced detection sensitivity. The optical response to acoustic excitation in the spectral region of Bragg’s law for the probe light possesses spectral peaks and nodes, the position of which can be controlled by the ANC parameters and probe wavelength.

## Methods

### Samples

The silica particles used for the self-assembly of the colloidal crystal thin films termed opals were synthesized via a modified Stöber method,^45^ as previously described in the literature.^46^ Opals are prepared using the vertical deposition method, where a glass substrate is immersed in a 0.1 wt % silica particle suspension, and the solvent is slowly evaporated over 3 days at 65 °C.

The vdW nanolayer transfer process uses a transfer stamp with polycaprolactone (PCL) similar to the method described earlier.^47^ WSe_2_ nanolayers are mechanically exfoliated onto a Si/SiO_2_ substrate and picked up with a PCL stamp. After contact with the opal film, the PCL is fully melted at >65 °C. This leaves the WSe_2_ nanolayers and a polymer residue on the opal surface after lifting the stamp. The PCL is then removed in a bath of tetrahydrofuran.

In the experiments, we measure 42 WSe_2_ nanolayers. The thicknesses *d* of the several layers are measured by AFM before the transfer on the opal layer. These measurements allow LA sound velocity to be obtained from eq 1, which is well-known for the fundamental breathing mode. For the major part of layers, *d* is obtained from eq 1 after measuring the frequency *f*_1_.

### Pump–Probe Technique

The laser ASOPS system used in the experiments is based on two TOPTICA lasers with fixed central wavelengths of 1050 and 780 nm. The second (525 nm) and first harmonics are used for the pump and probe excitations, respectively. The 80 MHz ASOPS system allows one to measure pump–probe signals in a 12 ns temporal interval and detect relative changes down to ∼10^–5^. Electronic triggering provides the temporal resolution ∼1 ps, which allows us to define accurately the oscillation frequencies up to 200 GHz. To measure the signals with low amplitude and better temporal resolutions (but a smaller temporal interval), it is required to use other systems (e.g., with fast delay lines^21^). It should be taken into account that ASOPS systems may give parasite oscillations due to the interference of laser beams. In our system, this results in a spectral line at *f* = 125 GHz (see FFT in Figure 2a).

The values of Δ*R*(*t*)/*R*_0_ are obtained by measuring the photoinduced electrical signals Δ*V*/*V*_0_, where Δ*V* and *V*_0_ are the ac and dc signals with and without pump excitation, respectively. Δ*V* and *V*_0_ are measured on the same Newport photodetector and the power of incident probe light is kept constant during these measurements. As a result, Δ*R*(*t*)/*R*_0_ = Δ*V*/*V*_0_.

### Calculations of Reflectivity Change

The equations for *C* and Ψ_*s*_(*t*) in eq 5 are presented elsewhere^40^ and may be written as
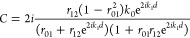
where *k*_*j*_ (*j* = 0, 1, 2) are the probe light wave vectors in the air (0), WSe_2_ nanolayer (1), and opal (2); *r*_*ij*_ = (*k*_*i*_ – *k*_*j*_)/(*k*_*i*_ + *k*_*j*_) are the reflection coefficients at the corresponding interfaces. The refractive index for opal is taken to be *N*_opal_ = 1.5. The photoelastic term in eq 5 includes integration of photon–phonon interaction inside the bulk of the WSe_2_ nanolayer
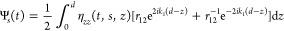


In the approximation, when the influence of carrier diffusion and recombination is negligible, the expression for the strain η_*zz*_(*t*, *s*, *z*) related with the *s*-th harmonic is given by eq 4.

### Estimation of Deformation Potential

The out-of-plane deformation potential for the exciton in multilayer WSe_2_ and other TMDs is known from neither experimental nor theoretical works. For the estimation, we use the data,^42^ where the exciton energy *E*_exc_ is studied experimentally in multilayer TMDs under hydrostatic pressure *P*. For WSe_2_, d*E*_exc_/d*P* = 3.4 meV/kbar. Assuming that the effect of out-of-plane stress dominates, we get Ξ = d*E*_exc_/dη_*zz*_ = −*v*^2^ρd*E*_exc_/d*P* ∼ – 3 eV. The value of Ξ is smaller if the assumption about the dominant out-of-plane effect for hydrostatic pressure is not valid. In Supporting Information 2, we plot the normalized dependences of *A*_1_(*f*_1_) for several values of Ξ from which we may conclude that we have a qualitative agreement between the experiment and theory for a wide range of negative Ξ, while the photoelastic contribution remains dominant. Finally, in the simulations, we use Ξ = −1 eV, which gives the best agreement for the dependence *A*_1_(*f*_1_).

## Supporting Information Available

Dependence of high harmonic amplitude on the spatial distribution of photogenerated stress; deformation potential for probe light (PDF)
