# Supplementary material for: Enhanced Photon–Phonon Interaction in WSe2 Acoustic Nanocavities
Source: ACS Photonics. 2024 Mar 7;11(3):1147–55. doi: 10.1021/acsphotonics.3c01601 (PMC10958595; doi:10.1021/acsphotonics.3c01601)
Supplement: Supplementary file 1 — ph3c01601_si_001.pdf [file ph3c01601_si_001.pdf]

Supporting information

Enhanced photon-phonon interaction in WSe_2 acoustic nanocavities

Alex D. Carr[1], Claudia Ruppert[2], Anton K. Samusev[2], Giulia Magnabosco[3], Nicolas Vogel[3], Tetiana L. Linnik[2,4], Andrew W. Rushforth[1], Manfred Bayer[2], Alexey V. Scherbakov[2], Andrey V. Akimov[1]*.

[1]School of Physics and Astronomy, University of Nottingham, Nottingham NG7 2RD, United Kingdom
[2]Experimentelle Physik 2, Technische Universität Dortmund, Otto-Hahn-Str. 4a, 44227 Dortmund, Germany
[3]Institute of Particle Technology, Friedrich-Alexander-Universität Erlangen-Nürnberg, Cauerstr. 4, 91058 Erlangen, Germany
[4]Department of Theoretical Physics, V.E. Lashkaryov Institute of Semiconductor Physics, 03028 Kyiv, Ukraine

1. *The dependence of high harmonic amplitude on the spatial distribution of photogenerated stress.*

In the main text of the paper the generated stress is assumed to decrease exponentialy with z (see eq 3). Actually, the spatial distribution of generated stress $G(t,z)$ is more complicated and governed not only by the absorption length of the pump excitation but also by the spatial diffusion of hot carriers and thermal heat. The parameters which are needed for taking these processes into account are not known but it is clear that spatial distribution of generated stress strongly influences the overlap with the generated acoustic modes, especially for higher harmonics, s >1. Here we consider a qualitative model where in the film of the thickness *d*, the generated stress is uniform within a depth $H \leq d$ (see Figure S1). We assume that for $z < H$ the temporal evolution of generated stress is the same as used in the main text $G(t,z) = DN_0\Theta(t)$ (see eq 3). In the rest of the film for $z > H$ the generated stress $G(t,z) = 0$.

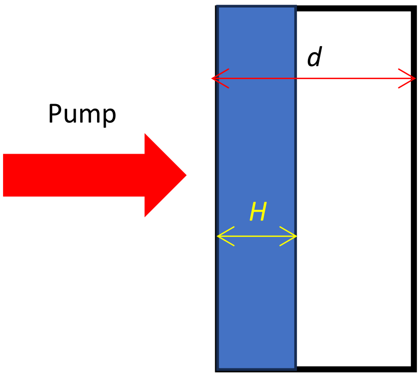

Figure S1. Scheme of the model for generated stress (blue rectangle) with a depth *H*.

The calculations for strain $\eta_{zz}(t,z) = \partial u/\partial z$ start with solving one-dimensional elastic equation (eq 2 in the main text) for the displacement $u(t,z)$ of atoms in the ANC:

$$\rho\frac{\partial^2 u}{\partial t^2} = \frac{\partial\sigma_{zz}(t,z)}{\partial z}, \quad \sigma_{zz}(t,z) = \rho v^2\eta_{zz}(t,z) + G(t,z)\,, \tag{S1}$$

with boundary conditions of zero stress at the free surfaces: $\sigma_{zz}(t,z) = 0$ at $z = 0$ and $z = d$, ρ =9.32 g/cm^3 is the mass density of WSe_2. The solution for the s-acoustic resonance in the considered model is:

$$\eta_{zz}(t,s,z) = \frac{DN_0}{\rho v^2}\frac{(-1)^{s+1}}{s\pi}[1 - \cos{(q_s H)}]\sin(q_s z)\, e^{i\omega_s t} \quad \text{(S2)}$$

Further the value of calculated stress is substituted to eq 5 presented in the main text for calculating the amplitude of reflectivity modulation for corresponding acoustic harmonic. The results of the calculations for three different values of H are shown in Fig. S2.

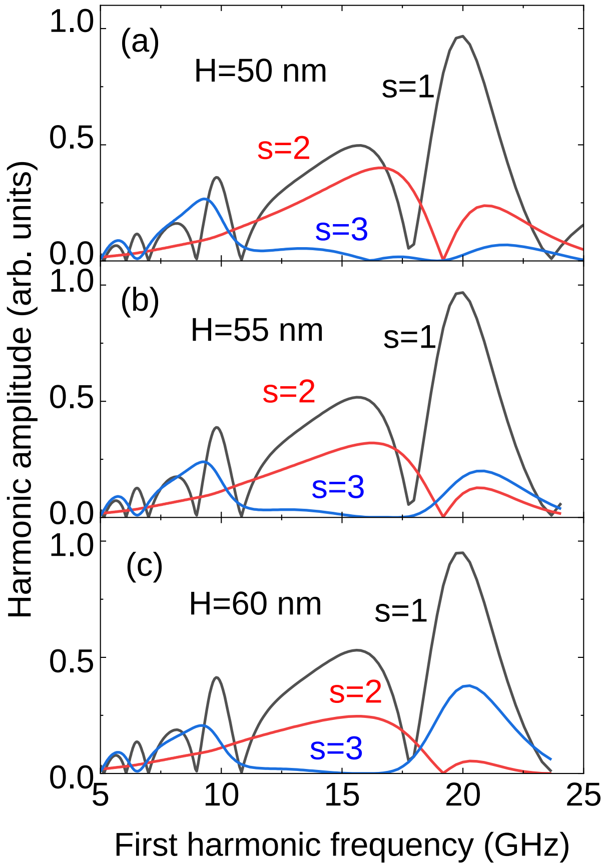

Figure S2. Normalized amplitudes of the first three harmonics of the temporal reflectivity signal as a function of the frequency of first harmonic (s=1) in the case of the uniform photogenerated stress for the three depths of the optically generated stress: H = 50 nm (a); 55 nm (b); and 60 nm (c). Different line colours correspond to s=1 (black); s=2 (red); and s=3 (blue).

One can see that the ratio of harmonic amplitudes depends strongly on the H. The relative amplitudes of the second and third harmonics relatively to the amplitude of the first

harmonic is smaller than in the theoretical curves in Figure 4 of the main text. In the experiments (see Figure 4) we also see that the amplitudes of 2nd and 3rd harmonics are a factor of two lower than the amplitude of the 1st harmonic. Basing on the qualitative analysis and comparison of Figures 4 (main text) and Figure S2 we may conclude that the spatial profile of the generated stress is more complicated than just the exponent governed by the penetration depth for pump light. More detailed analysis of the spatial profile of generated stress requires the knowledge of dynamical parameters for photoexcited carrier and heat.

2. *Deformation potential for the probe light*

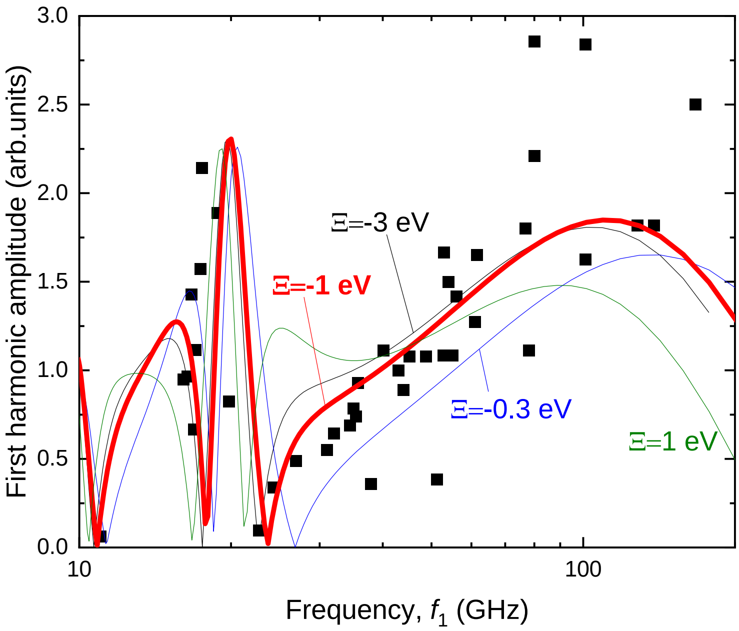

Figure S3. Dependence of the first harmonic amplitude on its frequency. The solid lines show the dependences calculated for different values of deformation potential Ξ for photon energy 1.6 eV (near the direct exciton resonance) in multilayer WSe_2. The value Ξ=-3 eV is the maximum possible value taken from the hydrostatic experiment [41]. The calculated curves are normalized for the frequency of photoelastic resonance (f_1 ≈20 GHz). The symbols are the experimental data (see Figure 4a of the main text). We find that the best qualitative agreement between the experiment and theory takes place for Ξ =-1 eV. The curves calculated for Ξ ≥0 do not fit the experimental data.
